# Oxidation Behavior of Multilayer Hard Coatings (TiCN/Al_2_O_3_/TiN) in Process of Recycling Coated Multicomponent Hardmetal Scrap

**DOI:** 10.3390/ma11101796

**Published:** 2018-09-21

**Authors:** Hai Kuang, Dunqiang Tan, Wen He, Zhiqiang Yi, Zhihang Zou, Xiaoru Wang

**Affiliations:** 1 School of Materials Science and Engineering, Nanchang University, Nanchang 330031, China; haizi411@126.com (H.K.); wshw82@163.com (W.H.); 18720990131@163.com (Z.Y.); zhihangzou@163.com (Z.Z.); 18270910065@163.com (X.W.); 2 School of Materials and Mechatronics, Jiangxi Science and Technology Normal University, Nanchang 330038, China

**Keywords:** recycle, TiCN/Al_2_O_3_/TiN coating, multicomponent cemented carbide scrap, oxidation resistance, cracks, diffusion

## Abstract

The coating is one of the biggest problems in the recycling of coated multicomponent hardmetal scraps. The isothermal oxidation behavior of WC-Co multicomponent cemented carbide inserts with a TiCN/Al_2_O_3_/TiN hard coating in the recycling process was investigated. The oxidation rate slowed down as the protective coating blocked element diffusion. A rapid oxidation rate was obtained when they were milled into powders and isothermally oxidized at 900 °C. A rapid path for element diffusion was provided by the defects, which were promoted by stress, expansion, and gas volatilization. Both the TiN and TiCN layers were oxidized to a porous TiO_2_ scale, while the Al_2_O_3_ phase remained and the dense Al_2_O_3_ layer acted as a barrier for its good oxidation resistance. Pieces of the Al_2_O_3_ layer were obviously seen in the final oxides. This provides critical information to reduce the negative effect of coatings and improve the performance of recycled WC powders and hard alloys.

## 1. Introduction

Tungsten is one of the most important metals and is widely used in the aerospace, industrial, and military applications for its excellent toughness and hardness, but it is subject to supply risk due to the limited deposits and increasing demand [1,2,3], and will soon be used up at the current consumption speed [4,5,6]. Therefore, it is of urgent need to recover tungsten resources. Recycling of tungsten-based cemented carbide is an important source of tungsten, which consumes less energy and requires low investment compared to the production of virgin powders. In fact, secondary tungsten had a share of 22% of global supply in 2014 and is increasing every year [7,8].

Only uncoated WC-Co hardmetal scraps such as cemented carbide anvils are popular for recycling. However, coated multicomponent cemented carbides are rarely recycled due to the additional coatings and mixed metal carbides in the substrate. Their complicated composition and structure make it difficult to obtain good recycled products because the coatings and different content of rare elements affect the recycling process. Meanwhile, additional elements such as Ti, Al, Ta, and Nb are left, which leads to the poor performance of recycled WC powders or hardmetals [9]. However, in recent years, researchers have focused on the large amount and relatively cheap coated multicomponent hardmetal scraps due to the sharply increasing price of uncoated scraps and the increasing demand of tungsten. Multicomponent cemented carbide inserts covered with titanium carbonitride (TiCN)/aluminum oxide (Al_2_O_3_)/titanium nitride (TiN) coatings are typical cutting tool tips. It is most widely used for its good oxidation resistance and thermal stability. The most common methods for recycling tungsten-based cemented carbides are the zinc melting method and hydrometallurgy. The zinc melting method has been reported for the recycling of WC-Co cemented carbide inserts with TiCN/Al_2_O_3_/TiN coatings. Though the thickness of the Al_2_O_3_ layer is usually below 2 μm, pieces of the Al_2_O_3_ coating have been found in recycled WC powders. High stability Al_2_O_3_ coatings neither decomposed nor reacted with WC or Co. It is also impossible to fuse when reclaimed powders are sintered to cemented carbides because of the high melting point (2054 °C). Thus, Al_2_O_3_ is defeated in recycled hardmetals, resulting in decreased adhesion strength in the interface of WC/Co, thus leading to poor performance in the recycled hardmetal. Moreover, the Al_2_O_3_ coating can also be left in Zn and repeatedly influence the recycled products [10]. On the other hand, the recycling of hardmetal scraps has been considered primarily based on hydrometallurgy [11]. However, a relatively long time and corrosive acid solution is required [4,12]. Furthermore, the refractory metal carbides in component cemented carbides such as TiC and (Ta,Nb)C undertake a partial replacement of WC during the recycling process. The various contents need different recycling parameters and it is difficult to unify, leading to unstable recycled products [13].

It is well known that the oxidation and reduction–carbonization process is a low-cost and environmentally friendly recycling method [14,15]. It usually contains three steps including oxidation, reduction, and carbonization reactions, or two processes of oxidation and carbothermal reduction. No corrosive solution is required and the process is short [16]. For TiCN/Al_2_O_3_/TiN-coated multicomponent hardmetals, the negative effect of the Al_2_O_3_-coating may be reduced since the Al_2_O_3_ may change to Al_4_C_3_ in carbonization reactions. It has been reported that the hardness only decreases a little when Al_4_C_3_ is present in cemented carbide [17]. Moreover, the TiN and TiCN coatings may transform to the TiC phase, while the TaC, TiC, and NbC in the substrate remain in the original chemical phase. The new carbides act as strengthening phases in the recycled hard alloy, improving hardness and strength. Oxidation is the most important procedure, and determines the recovery rate and the transformation of coatings as well as the metal carbides and Co. However, up until now, the scientific literature reporting on the oxidation behavior of multicomponent cemented carbides with TiCN/Al_2_O_3_/TiN coatings when recycled has been rare. Understanding this is vital for the optimal oxidation process and even for the quality of the final recycled products.

In this work, representative multicomponent WC-Co-(TiC, TaC, and NbC) cemented carbide inserts with a TiCN/Al_2_O_3_/TiN coating were oxidized in air. The aims were to reveal the oxidation behavior, focusing on the influence of coatings on the oxidation rate, phase transformation, and evolution of TiCN/Al_2_O_3_/TiN coatings. This is crucial to the subsequent reduction and carbonization reaction as well as the final recycled hardmetals.

## 2. Materials and Methods

### 2.1. Materials

**Figure 1:**
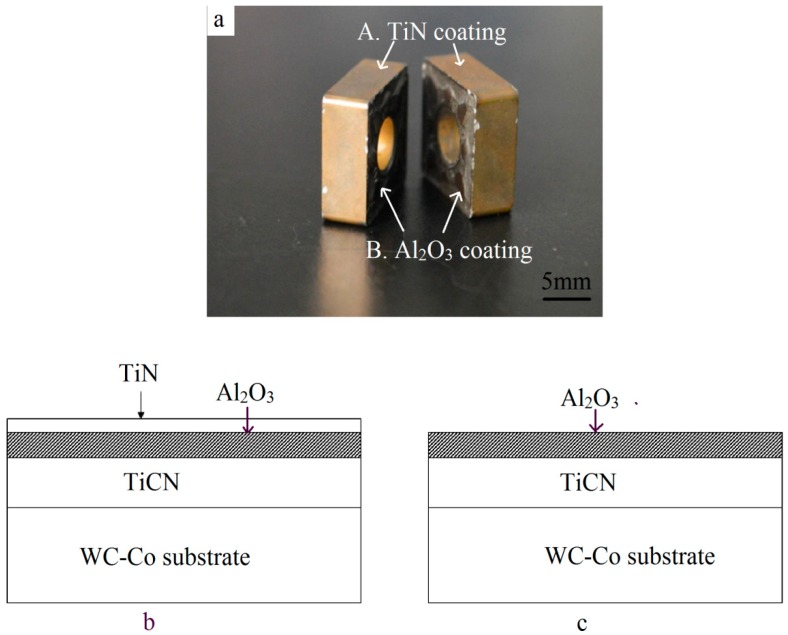
Multilayer coating on the multicomponent substrate. (**a**) Macrograph; (**b**) schematic of the TiCN/Al_2_O_3_/TiN coating on four flanks; and (**c**) schematic of the TiCN/Al_2_O_3_ coating on the two front surfaces.

Discarded turning tool tips were pursued for the raw materials (Figure 1a). The dimensions were 12 mm × 12 mm × 5 mm and their weight was about 9 g. As shown in Table 1 [18], the main chemical composition was WC-7.41% Co, and also contained a small number of other elements such as Ta, Ti, and Nb for good performance. The insert was covered with a TiCN/Al_2_O_3_/TiN coating (Figure 1b) through the chemical vapor deposition (CVD) method, while the other two faces were covered with TiCN/Al_2_O_3_ (Figure 1c) due to the removal of the outermost TiN layer for less stress. Due to the repetitive machining operations, localized areas of the coating began to disintegrate locally, especially at the corners and edges (Figure 1a).

### 2.2. Experimental Procedures

**Figure 2:**
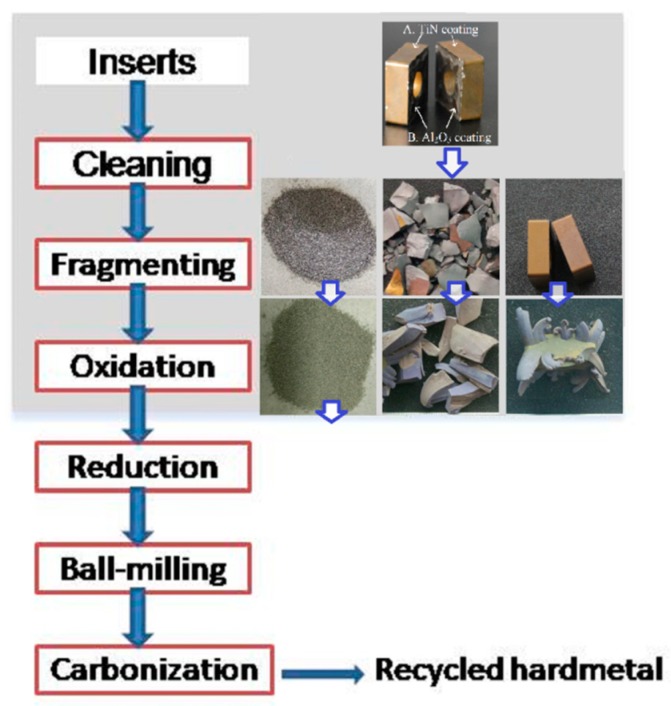
Recycling of coated multicomponent cemented carbides by the oxidation and reduction–carbonization process.

Figure 2 shows the recycling process for the coated multicomponent cemented carbide by the oxidation and reduction–carbonization reaction. The treatments in the light-grey square area were performed in this paper. Worn inserts were ultrasonically cleaned with acetone and ethanol successively. Then, the cleaned samples were dried sufficiently in a drying oven. Some of these were manually fragmented into pieces or milled into powders. The untreated samples and the fragments were put into alumina crucibles and isothermally maintained in air at 720 °C and 900 °C in electrical furnaces (SX2-4-10, Yifeng, Shanghai, China), with holding times of 10, 30, 60, 120, 180, and 300 min. The milled powders were sieved passed through a 100-mesh screen to ensure the formation of fine and uniform products, and then isothermally heated in air at 900 °C for 180 min.

Thermal analysis was conducted by thermogravimetric and differential scanning calorimetry (TG–DSC) (NETZSCH-STA449. F5, Netzsch, Selb, Germany) to detect the dynamic oxidation behavior of the samples with a heating rate of 10 °C/min in the range of 25~1100 °C under air flow. The size of the coated WC-Co particles for the TG–DSC was below 0.15 mm and the weight was less than 10 mg due to the limitations of the equipment. The oxidation weight was tested at room temperature by an electronic balance (Lucky, Wuxin, Yongkang, China) with an accuracy of 0.001 g. Backscattered electron (BSE) morphologies of the samples oxidized at different times were tested by the field emission scanning electron microscope (FESEM, Nova NanoSEM450, FEI, Hillsoro, OR, USA) with a voltage of 20 Kv under vacuum, whilst the element compositions were characterized by energy-dispersive X-ray spectroscopy (EDS, X-max 80, Oxford Instruments, Oxford, UK). Chemical phases were identified by X-ray diffraction (XRD, PANalytical, EMPYREAN, Almelo, Netherlands) in the Bragg–Brentano geometry with Cu-Kα radiation (λ = 0.154 nm) under a voltage of 40 KV and current of 40 mA. The diffraction patterns were obtained using the continuous scanning mode in 2θ ranging from 10° to 90° with a step size of 0.01° and a speed of 2°/min. The average thickness of the coatings and width of cracks were obtained by taking measurements accordingly from the SEM images using image analysis software (NanoMeasurer1.2, Fudan University, Shanghai, China).

## 3. Results and Discussion

### 3.1. Oxidation Kinetics

**Figure 3:**
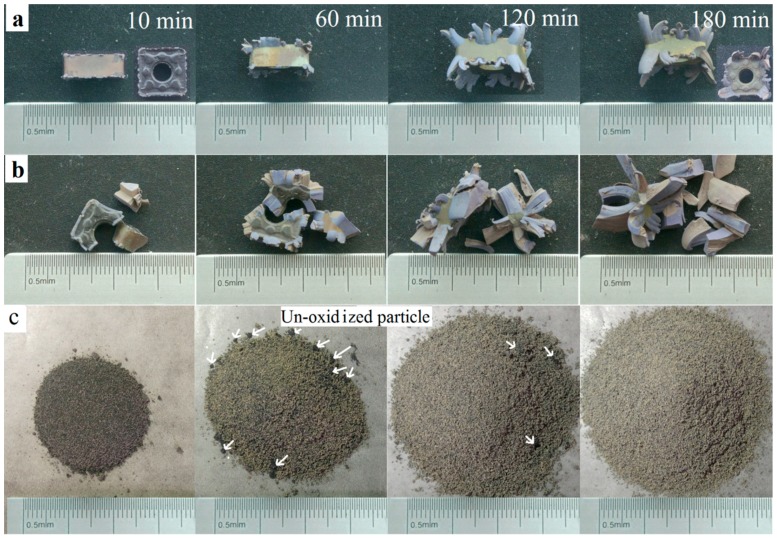
Macrographs of samples oxidized at 900 °C in air for different times. (**a**) Untreated samples; (**b**) fragmented samples; and (**c**) milled powders.

Coated inserts were oxidized at 900 °C in air for different times. Oxidation can be fully finished at the temperature of 900 °C according to previous reports [4,5]. Macrographs represented in Figure 3. At the early stage, the compact coating acted as an effective diffusion barrier, blocking the inward diffusion of oxygen and the outward diffusion of the elements inside. After being oxidized for 10 min, the coating was still smooth. Only a few blue-gray oxides could be seen upwardly bulging from the edges and corners, where the defects were concentrated (Figure 1a). Defects provided a rapid path for oxygen to diffuse into the substrate. Oxidation was initiated from the damaged edges and corners, especially the local loss of the protective coating (Figure 3). More bulges can be found with further oxidation. When oxidized for 60 min, the color of the outermost layer becomes uneven. After oxidation for 120 min, it could be seen that some bulges came out of the middle of the coating on the flank faces, not only on the edges. The front faces were a little yellow when oxidized for 180 min. However, the untreated samples approximately remained as a square shape and were only partially oxidized for 180 min (Figure 3a). This was different from the result reporting that the CrAlN coated WC-9% Co insert tip scraps were fully oxidized at 900 °C for 180 min [12]. This discrepancy can be attributed to the better oxidation resistance of the Al_2_O_3_ coating to CrAlN. Therefore, different coatings should be given a different treatment.

Figure 3b shows the macrographs of the fragments with different oxidation times. As shown in pictures, when oxidized for 10 min, the shape changed considerably. Swelling was visible at the locations of coating loss where the oxidation reaction was not limited by the coatings. Oxidation continued individually at many of the different damaged locations, so the shape was irregular. When undergoing oxidation for 180 min, the volume was up to more than 200%. The result revealed that the oxidation rate was apparently accelerated when the hardmetal scrap was broken into pieces since more bare WC-Co phases could come into contact with the oxygen directly.

Figure 3c shows the milled powders that were isothermally heated at 900 °C in air for various times. When oxidized for 10 min, many black particles could be seen, and the quantity of the particles decreased with further oxidation. The powders became a completely light grey-green in color after oxidation for 180 min and no black un-oxidized particles were found, indicating that the milled powders were fully oxidized after oxidation treatment for 180 min. Compared to the untreated inserts and fragmented samples, the oxidation rate greatly accelerated after the milling procedure.

**Figure 4:**
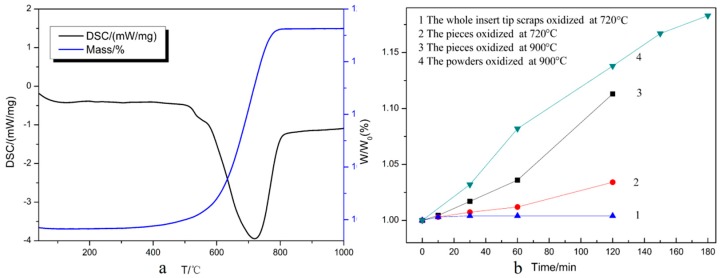
(**a**) TG-DSC curves of the coated multicomponent hardmetal oxidized under air flow; and (**b**) weight ratios versus oxidation time.

To examine the optimal oxidation temperature, thermal analysis was employed with air flow. The TG-DSC results are shown in Figure 4a. The mass fraction indicates the total weight of the oxidized samples as a percentage compared to the initial weight. It can be seen that the weight changed obviously with increasing temperature after 400 °C and increased sharply around 720 °C. This means that oxidation began at around 400 °C, so the theoretical oxidation temperature for recycling the WC-Co cemented carbide scrap should be 720 °C. As shown in Figure 4a, the TG curve remained almost flat when the value of the mass fraction reached 118.20%, which as consistent with the theoretical calculation value when fully oxidized, indicating that the weight gain was about 18.20% when fully oxidized [19].

Figure 4b shows the relationship between the weight ratio *W/W*_0_ and oxidation time. *W* is the total weight of the oxidized samples compared and *W*_0_ is the initial weight. According to the result in Figure 4a, the coated inserts could be rapidly oxidized at 720 °C. However, the curve appeared flat when the untreated hardmetal scraps were oxidized at 720 °C (Curve 1), i.e., the whole insert tip scrap could not be completely oxidized at 720 °C since the coating strongly resisted the oxidation. It has been reported that the oxidation process is controlled by the reaction at the interfaces [19]. For the untreated samples, the WC-Co surfaces were separated from oxygen by the coating, and as the oxygen could not directly make contact with the inner WC-Co phase, the oxidation rate slowed down for the protective coatings. In order to accelerate the oxidation process, it was necessary to reveal more bare WC-Co phases to oxygen, thus fragmentation was conducted. The weight ratio still increased slowly when at 720 °C (Curve 2), so a higher temperature was required. When the oxidation temperature rose to 900 °C, an obvious weight increment could be observed and the oxidation rate was significantly accelerated (Curve 3). This revealed that fine particles and increasing oxidation temperature led to an increasing oxidation rate. Coated inserts were milled to powders with a size below 0.150 mm. Thus, nearly all of the WC-Co surfaces were exposed to air, just like the uncoated particles. Oxygen could therefore make contact with the WC-Co surfaces directly and reacted with them. Furthermore, the oxygen also could easily diffuse into the WC-Co surfaces near the coating so the weight ratio increased markedly and reached 1.138 when oxidized for 120 min. With further oxidation for 180 min, it reached 1.183, approximate to the max mass value of 1.182 in Figure 4a. This implies that the powders that had been passed through a 100-mesh sieve were fully oxidized at 900 °C for 180 min. The results demonstrated that the coating slowed down the oxidation rate. Considering the cost and production cycle, the scrap should be milled into powders for rapid processing.

### 3.2. Phase Transitions

**Figure 5:**
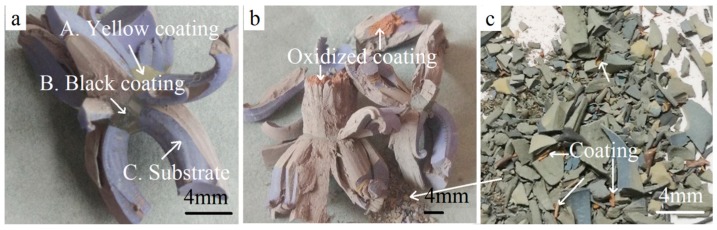
Magnified macrographs of fragments oxidized at 900 °C for different holding time. (**a**) 180 min; (**b**,**c**) 300 min.

Figure 5 shows the enlarged macrographs of the coated WC-Co hardmetal scraps oxidized at 900 °C in air for 180 min and 300 min. It can be observed from Figure 5a that the yellow coating and black coating connected to the substrate when oxidized for 180 min. This implies that the TiCN/Al_2_O_3_/TiN and TiCN/Al_2_O_3_ coatings were not fully oxidized. With further oxidization for 300 min, red porous oxides were seen clearly in Figure 5b,c, indicating the phase transition. Bulges were porous and could be easily broken down for the cracking of the hard phases and bond metals during oxidation treatment [19].

**Figure 6:**
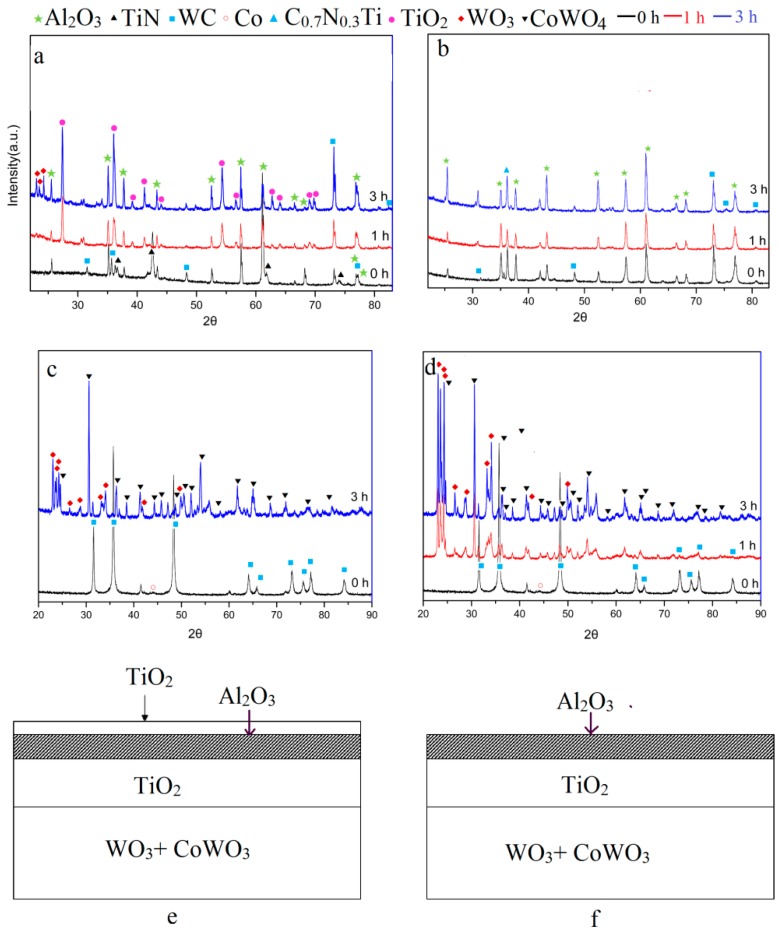
Chemical phases. XRD pattern of fragments oxidized for different oxidation time (**a**) the outmost yellow coating in flanks, (**b**) black coating in front faces, (**c**) substrate, (**d**) the XRD pattern of the fully oxidized powders, schematic diagrams of fully oxidized fragments (**e**) coated with yellow TiCN/Al_2_O_3_/TiN layers, and (**f**) coated with black TiCN/Al_2_O_3_ layers.

Figure 6 shows the chemical phases of the fragments oxidized for different times. Figure 6a presents the XRD patterns of the outermost yellow TiN coating. When oxidized for 60 min, the TiO_2_ (PDF#21-1276) phase appeared. The result showed that the yellow coating that was oxidized mainly consisted of the TiO_2_ phase. TiO_2_ was the final oxidation product of the TiN coating as well as TiCN. This can be described by the reactions as [20].

2TiN + 2O_2_ → 2TiO_2_ + N_2_ (1)

2TiCN + 4O_2_ → 2TiO_2_ + N_2_ + 2CO_2_ (2)

On the other hand, the black coating was mainly composed of the Al_2_O_3_ (PDF#46-1212) phase as originally based on the XRD pattern in Figure 6b. The chemical compound Al_2_O_3_ is impossible to oxidize due to its good oxidation resistance at 900 °C [20]. Therefore, the phases were almost the same with different oxidation times.

Figure 6b,c show the XRD results of the substrate and milled powders oxidized for different oxidation times. It was found that the peaks of WC (PDF#51-0939) decreased with further oxidation. After oxidation for 180 min, the present coated WC-Co hardmetal powders were subsequently transformed into a mixture of WO_3_ (PDF#20-1324) and CoWO_4_ (PDF#15-0867) as well as the exposed substrate. The following oxidation reactions could be proposed [15,19].

WC + 5/2O_2_ → WO_3_ + CO_2_ (3)

WC + 2O_2_ → WO_3_ + CO (4)

Co + WC + 3O_2_ → CoWO_4_ + CO_2_ (5)

In accordance with the reported thermodynamic calculations [12,14], the standard Gibbs free energy of reactions (Equations (3)–(5)) were all below zero and the reactions took place under standard pressure at 900 °C. This was demonstrated by the XRD result of the substrate surface in Figure 6a. When the WC-Co hardmetal scrap covered with the TiCN/Al_2_O_3_/TiN multilayer was fully oxidized, it could be seen as the schemes shown in Figure 6e,f, and the unbroken oxidized layer could be TiO_2_/Al_2_O_3_/TiO_2_. In fact, it was difficult to keep the whole shape as the brittle oxide layers and porous substrates were easily broken down.

After oxidation for 180 min, no un-oxidized WC peaks were detected in the powders (Figure 6d), but could be found on the surface of the fragments (Figure 6a,b), i.e., the milled powders passed through a 100-mesh were fully oxidized, but the bulks were not. This contradiction can be explained by the protective coatings. Therefore, the crushing process should be done before oxidation for a shorter process, which is meaningful for reducing energy consumption.

The intensity peaks caused by TiO_2_ and Al_2_O_3_ were not detectable in the oxide powders due to their relatively low content as well as Ta_2_O_5_ and Nb_2_O_5_, which are the oxides of Ta and Nb, respectively [13,21]. It was speculated that their peaks were hidden by the XRD pattern noise given their weak diffraction signals. The possible chemical reactions and oxidation behavior of TaC, NbC, and TiC were the focus of our study and will be discussed in our future publication.

**Figure 7:**
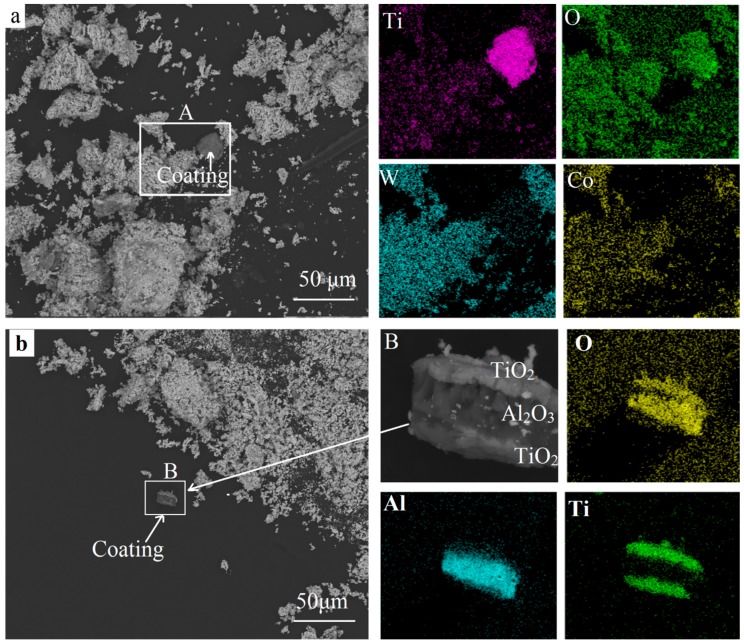
SEM images and the EDS results of the recycled oxides. (**a**) gray coating in final oxides; (**b**) multlayer coatings in final oxides.

Figure 7 shows the SEM images and the EDS results of the fully oxidized powders. According to the XRD patterns in Figure 6d, the final oxides were mainly consistent with WO_3_ and CoWO_4_. This agreed well with the results reported [4,5]. As shown in Figure 7a, a gray fragment could be seen clearly among the white final oxides in the square area A. This fragment was rich in titanium and oxygen, while tungsten and cobalt were concentrated in the white particles. This could be the exfoliation of the TiO_2_ layer according to the XRD result in Figure 6a. Pieces of the multilayer could be found in area B in Figure 7b where oxygen was focused. Aluminum was concentrated in the middle layer, while titanium was distributed on the inner and outermost layers. Based on the XRD results in Figure 6, this could be the TiO_2_/Al_2_O_3_/TiO_2_ layers. Therefore, the TiO_2_ and Al_2_O_3_ phases remained in the oxide powders. Although the Al_2_O_3_ layer was thin, it was difficult to fuse or react with the WC and Co. It mainly distributes in the binder Co and decreases the bonding strength of WC/Co [17]. Future work will focus on the influence of Al_2_O_3_ coatings on the subsequent reduction and carburization processes as well as the recycled products of the W powders, WC-Co composite powders, and the final recycled hard alloy.

### 3.3. Evolution of Coatings

**Figure 8:**
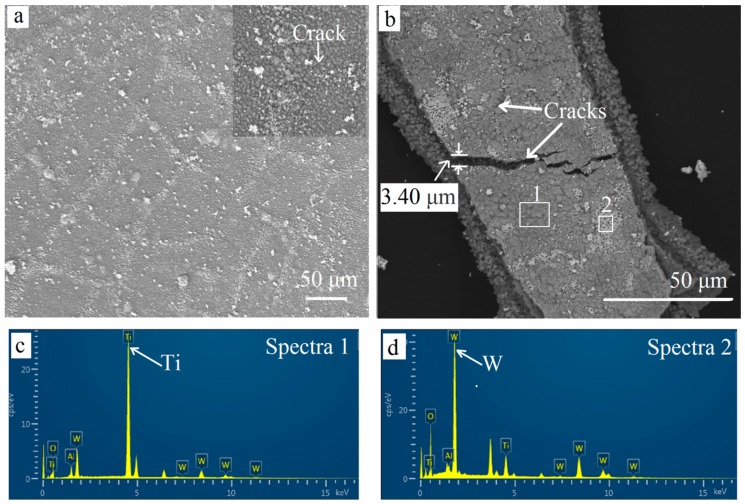
Surface SEM images for different oxidation times. (**a**) 60 min; (**b**) 300 min. EDS results (**c**) oxidized layers; (**d**) white particles on surface.

**Figure 9:**
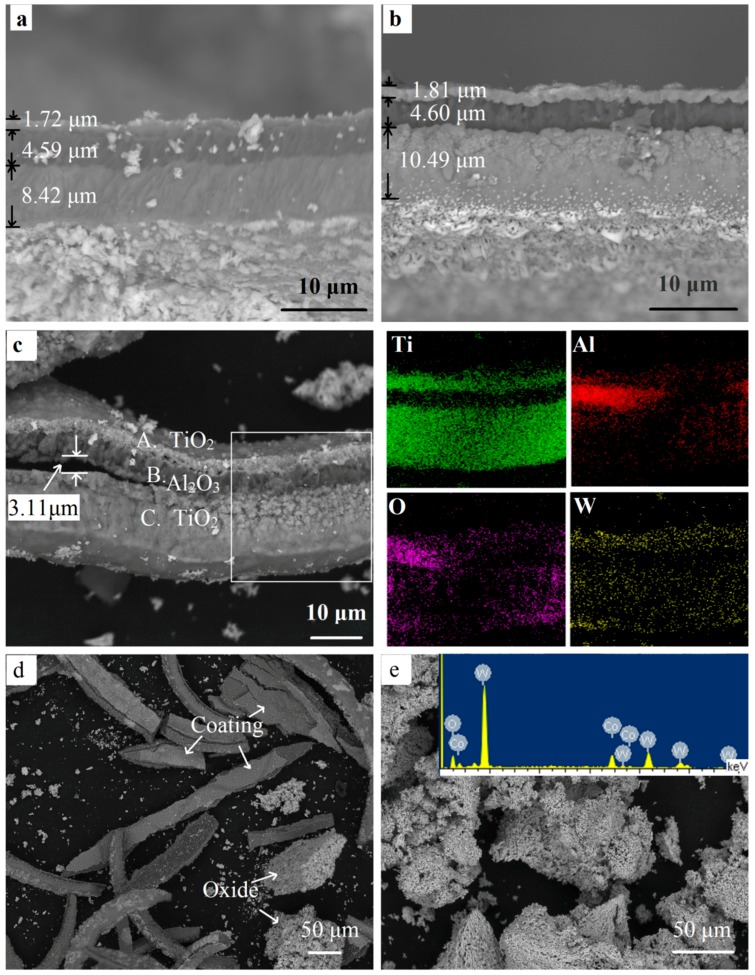
The fractured cross-sectional SEM images and the EDS results of the fragmented coated WC-Co hardmetal scrap at different oxidation times. (**a**) 10 min; (**b**) 30 min; (**c**–**e**) 300 min.

To understand the development of the coated insert and the interaction between oxygen, the coating, and substrate during oxidation, the morphologies of the samples were tested, as shown in Figure 8 and Figure 9. Before oxidation, many pits could be seen on the surfaces, and were concentrated at the corners and edges. These defects mainly appeared during the repetitive high-speed cutting operation, with even some bare WC-Co surfaces exposed to the local loss of coatings (Figure 3a). Stress was another main reason for the pits and micro-cracks. When cutting metals or alloys, the temperature in the inserts increased, then stress was generated for the different coefficients of thermal expansion (Table 2 [22]).

After oxidation for 60 min, more pits were found on the surface (Figure 8b). The outermost coating became uneven and porous. Furthermore, many cracks were presented and could be seen clearly in the magnified image on the left. The stress that appeared when isothermally heated at 900 °C was the main reason for more defects. Defects provided fast paths for the element diffusion. Oxygen diffused to the inner TiCN coating and inside the substrate through cracks and pits. Meanwhile, the elements inside were out-diffused. Some white oxide particles were piled up on the surface along the cracks. Some cracks were even healed by the white particles. After oxidation for 300 min, more and deeper cracks could be found. The widest crack exceeded 3 μm (Figure 8b). As shown in the EDS results (Figure 8c,d), the elements W was tested in the white particles and implies that W out-diffused to the surface.

Figure 9 shows the cross-section images and EDS results forth fragmented samples. Most coatings remained compact and protective after oxidation for 10 min (Figure 9a). After a mere 30 min of oxidation at 900 °C, the inner and outermost layers obviously expanded, especially for the inner coating where the thickness increased by 24.58% (Figure 9b). A number of pores and voids could be seen. Lofaj [19] suggested the presence of oxides and volatile gas as the main reason for the extensive swelling. As shown in Reactions (1) and (2), the TiN and TiCN layers were transformed to porous TiO_2_, accompanying the formation of volatile gas N_2_ and CO_2_. The volume expansion and growth of defects were caused and promoted by the presence of volatile gas as well as the volatile tungsten oxides [19,23]. As a consequence, the oxide particles in the porous inner and outermost layers were weakly bonded with each other, so that they were easily separated.

As the coating became un-protective, more oxygen moved into the inner TiCN coating and WC-Co substrate rapidly through these defects. The adhesion between the coatings became poor. As shown in Figure 9c, after a holding time of 300 min, the coating was highly cracked. A fissure with a maximum width exceeding 3 μm could be observed lying between the oxide layers. This indicated poor interfacial adhesion, which meant that the coating was easily broken down, resulting in the exfoliation of the coatings, as shown in Figure 5 and Figure 9d. Therefore, poor interfacial adhesion and defects are the main reasons for the breakdown of coatings [24]. Figure 9e shows the oxides when the powder sample was oxidized for 300 min. From the EDS result, there no C element could be found, only W, Co, and O were detected, indicating that the hardmetal was fully oxidized, and the stripped coating can be clearly seen in Figure 9d. This was in good agreement with the result in Figure 7. The coatings were distributed unevenly in the final oxides.

As seen in the EDS results of the square area in Figure 9c, the coating included three distinct layers. The outermost and inner layers were rich in titanium and oxygen. It confirmed the fact that both TiN and TiCN transformed to the TiO_2_ phase when the holding time was extended to 300 min. Although part of the area was hidden, Al could be seen to be concentrated in the middle layer, where there was an O element enrichment area. This indicates that the chemical phase Al_2_O_3_ remained, which was confirmed by the XRD pattern of the coatings. The dense Al_2_O_3_ coating acted as a barrier layer and the elements could hardly pass through it [20]. The outward diffusion of tungsten and the inward diffusion of oxygen mainly went through the defects, which are vulnerable, providing the most preferential and fast diffusion paths. Therefore, it can be seen that tungsten was distributed evenly in the outermost layers (Figure 9c) and on the surface of the samples (Figure 8c,d). This was confirmed by the WO_3_ peaks in the XRD results of the yellow coating in Figure 6a. However, only a little white WO_3_ could be observed, ascribing to a much more rapid in-diffusion of oxygen by gas phase diffusion as compared with the out-diffusion of W by solid state diffusion [25].

## 4. Conclusions

The isothermal oxidation of TiCN/Al_2_O_3_/TiN-coated WC-Co cemented carbide inserts with mixed carbides (TiC, TaC, NbC) was investigated as one of the main processes for recycling the coated multicomponent hardmetal scraps. The oxidation rate was reduced because the coating blocked the element diffusion. The fragments exhibited obvious weight increment at 900 °C when compared to the untreated sample. Milled powders passed through a 100-mesh sieve quickly transformed to a mixture of WO_3_ and CoWO_4_. The oxidation rate improved as more WC-Co phases were not limited by coatings after crushing and milling. For mechanical damage and stress during the cutting operation, many pits were concentrated at the corners and edges. During oxidation, defects appeared and were promoted by stress, volatilization, and swelling. Defects provided rapid paths for the in-diffusion of oxygen and the out-diffusion of elements inside.

Upon oxidation, both the TiN and TiCN coatings reacted with oxygen and quickly transformed to a porous TiO_2_ scale. The Al_2_O_3_ coating remained dense and acted as a barrier layer, blocking element diffusion. Oxygen moved inside mainly through the pores, fissures, and cracks. Pieces of Al_2_O_3_ and TiO_2_ layers were found in the recycled oxides.

This paper provides critical information to solve the problem of recycling coated hardmetals, especially with the Al_2_O_3_ coating. Reducing the negative effect of coatings on recycled hard alloys or even making use of Al_2_O_3_ to be reinforcement phase to improve the performance of recycled hardmetal will be the focus of study in the future. This is the most important factor for the recovery of tungsten resources from the great number of coated multicomponent cemented carbide scraps.

## Figures and Tables

**Table 1:** Main chemical composition of the coated multicomponent hardmetals.

Element	Co	W	C	Ta	Ti	Nb	Others
W/%	7.41	81.48	5.91	2.69	1.96	0.43	0.12

**Table 2:** Thermal expansion coefficients of materials.

Material	TiCN	TiN	Al_2_O_3_	WC
Thermal expansion coefficient (10^−6^ K)	7.80	9.35	9.00	4.30
